# Prediction of Severe Acute Pancreatitis Using a Decision Tree Model Based on the Revised Atlanta Classification of Acute Pancreatitis

**DOI:** 10.1371/journal.pone.0143486

**Published:** 2015-11-18

**Authors:** Zhiyong Yang, Liming Dong, Yushun Zhang, Chong Yang, Shanmiao Gou, Yongfeng Li, Jiongxin Xiong, Heshui Wu, Chunyou Wang

**Affiliations:** 1 Pancreatic Disease Institute, Department of General Surgery, Union Hospital, Tongji Medical College, Huazhong University of Science and Technology, Wuhan, Hubei Province, People's Republic of China; 2 Organ Transplantation Center, Hospital of University of Electronic Science and Technology of China and Sichuan Provincial People's Hospital, Chengdu, Sichuan, People's Republic of China; University of Szeged, HUNGARY

## Abstract

**Objective:**

To develop a model for the early prediction of severe acute pancreatitis based on the revised Atlanta classification of acute pancreatitis.

**Methods:**

Clinical data of 1308 patients with acute pancreatitis (AP) were included in the retrospective study. A total of 603 patients who were admitted to the hospital within 36 hours of the onset of the disease were included at last according to the inclusion criteria. The clinical data were collected within 12 hours after admission. All the patients were classified as having mild acute pancreatitis (MAP), moderately severe acute pancreatitis (MSAP) and severe acute pancreatitis (SAP) based on the revised Atlanta classification of acute pancreatitis. All the 603 patients were randomly divided into training group (402 cases) and test group (201 cases). Univariate and multiple regression analyses were used to identify the independent risk factors for the development of SAP in the training group. Then the prediction model was constructed using the decision tree method, and this model was applied to the test group to evaluate its validity.

**Results:**

The decision tree model was developed using creatinine, lactate dehydrogenase, and oxygenation index to predict SAP. The diagnostic sensitivity and specificity of SAP in the training group were 80.9% and 90.0%, respectively, and the sensitivity and specificity in the test group were 88.6% and 90.4%, respectively.

**Conclusions:**

The decision tree model based on creatinine, lactate dehydrogenase, and oxygenation index is more likely to predict the occurrence of SAP.

## Introduction

Acute pancreatitis (AP) is a common acute abdominal disease with an annual incidence of 13–45 cases per 100,000 populations [1–3]. The overall mortality is 2–5% [1, 2], and the mortality of patients diagnosed with severe acute pancreatitis (SAP) according to the 1992 Atlanta classification was as about 10–30% [4, 5]. Due to the high heterogeneity of the disease, patients with mild acute pancreatitis (MAP) only require fasting and fluid infusion treatment without excessive medical intervention, and patients will recover within one week or so. However, for critically ill patients, there are great differences in treatment for patients with different local complications and organ dysfunctions. To provide a better description of the severity of AP, a revised Atlanta diagnostic criteria guideline was issued in 2012 [6] following a previous issue in 1992 [7]. According to the new revised Atlanta diagnostic criteria guideline 2012, AP is classified as MAP, moderately severe acute pancreatitis (MSAP), and SAP. The length of hospital stay, intensive care unit stay time, mortality show significant differences among the three groups [8, 9], indicating that the new criteria can more accurately describe the severity of the disease, particularly identify critical ill patients. However, the classification of acute pancreatitis using the new criteria can only be obtained after observing the patients for 48 h or an even longer time. Previously, Ranson’s criteria and the Acute Physiology and Chronic Health Evaluation II scores were used to represent the disease severity and have been widely used by clinicians. Whereas, these two diagnostic criteria require a large amount of clinical data to be obtained within 24 to 48 h to make a conclusion. Single indicators such as blood glucose [10], pleural effusion [11], and C-reactive protein (CRP) [12] are also used to predict the severity of diseases, which greatly simplified the evaluation process; However, these indicators have poor sensitivity and specificity. Hence, in the present study, it was intended to find a measure that can quickly, easily, and more accurately predict SAP according to the revised Atlanta 2012 classification.

The decision tree analysis is a nonparametric statistical method, and its results are illustrated using a tree structure. The decision tree model illustrated by a flowchart is in agreement with the clinical data and is easily to be accepted by clinicians; thus, it has been used in the prediction models of different diseases [13,14]. In the present study, the clinical data of the patients within 12 h of admission were analyzed to develop a decision tree model to predict SAP according to the revised Atlanta classification criteria 2012.

## Materials and Methods

The study protocol was approved by the Ethics Committee of Union Hospital Tongji Medical College, Huazhong University of Science And Technology. All involved patients provided informed written consent before the start of any treatment. In this retrospective study, patients (n = 1308) with acute pancreatitis who were admitted to the Department of Pancreatic Surgery, Union Hospital, Tongji Medical College, Huazhong University of Science and Technology between January 2008 and June 2013, were included. Diagnostic criteria for acute pancreatitis accorded with two or more of the following three criteria: (1) sudden abdominal pain; (2) levels of serum amylase or lipase that were greater than three times the upper limit of normal range; and (3) imaging studies revealing peripancreatic exudation or pancreatic/peripancreatic necrosis [15]. Among these patients with acute pancreatitis, 603 patients met the inclusion criteria mentioned below, and they were included in this retrospective analysis: (1) patients who were admitted within 36 h of the onset of the disaese; (2) patients aged older than 18 years; (3) patients with no history of pancreatitis; and (4) patients with no history of cardiac failure, respiratory dysfunction, or renal failure. According to the revised Atlanta classification of acute pancreatitis 2012, patients were categorized into three groups: MAP, MSAP and SAP. The criteria for organ failures were as follows: (1) respiratory failure: oxygenation index (OI) smaller than 300; (2) renal failure: a serum creatinine level greater than 170 μmol/l or 1.9 mg/dl; and (3) cardiac failure: systolic blood pressure (SBP) less than 90 mmHg, and no response to fluid resuscitation. Patients who had organ failure for more than 48 h were classified as having SAP. Patients who had organ failure for less than 48 h or had local complications were classified as MSAP. Patients without organ failure as well as without local complications were classified as MAP [6]. Local complications were identified by retrospectively analyzing the imaging of contrast enhanced CT scans during hospitalization of the patients, which included the presence or absence of acute peripancreatic fluid collection, pancreatic pseudocysts, acute necrotic collections, and walled-off pancreatic necrosis. Almost every patient underwent contrast enhanced CT scans 72h after the symptom onset and repeat this examination every one week in severe patients. The OI was calculated by PaO_2_/FiO_2_. The PaO_2_ was measured in arterial blood gas analyses and the FiO_2_ was calculated by the oxygen flow rate. Room air was calculated as 21%, 2L/min of oxygen flow rate was calculated as 25%, 4L/min of oxygen flow rate was calculated as 30%, 6-8L/min of oxygen flow rate was calculated as 40%. Arterial blood gas analyses was performed only when the patients had a sign of respiration organ failure, such as the respiratory rate (RR) was more than 26pbm and the SpO_2_ detected in the finger was as low as 90%. The main purpose of this study was to distinguish SAP in the early disease stage; hence, MSAP and MAP were considered as non-SAP groups.

Clinical data, including the patients’ gender, age, chemical examination results, and monitoring indicators within 12 h of admission, including body temperature, pulse, blood pressure, RR, white blood cell count, platelet count, hematocrit, glucose, creatinine, blood urea nitrogen (BUN), and electrolyte levels, were collected (S1 File).

All patients with AP were managed according to United Kingdom and International Association of Pancreatology guidelines, as well as guidelines of the Chinese Society of pancreatitis [16, 17].

## Statistical Analysis

Continuous variables were expressed as the mean ± standard deviation, and data analyses were performed using Empower(R) (www.empowerstats.com, X&Y Solutions, Inc., Boston, MA, USA) and R (http://www.R-project.org) software. The classification model was developed using the classification and regression tree (CART) analysis and SPSS software, Version 18.0. A *P* value less than 0.05 was considered statistically significant.

The patients (n = 603) were randomly divided into the training group (n = 402) and test group (n = 201) with a ratio of 2:1 by using the computer random number generator. T-test and chi-squared tests were used to assess each indicator in the training and test groups. The results showed that each indicator had no statistically significant difference between the two groups. Next, the decision tree model of SAP in the training group was developed using logistic regression analysis, and each indicator with P<0.05 was included in the multiple regression analysis to further identify the independent risk factors and calculate the odds ratios (ORs) and 95% confidence intervals (CIs).

The decision tree analyses were performed using the obtained independent risk factors. We set the depth of the decision tree is no more than 3, so it will only include 3 factors to analysis. The parent node was set more than 20 if it will be regroup by a condition and the child node was set more than 1 for there is no need to regroup. All the cut-off points of the variables using for regroup the patients were obtained by the decision tree program using SPSS 18.0 software. The best decision tree model was identified based on the sensitivity and specificity of the prediction. After the satisfying model was gender in the training group, the model was tested again in the testing group.

## Results

### Randomization and general population

The patients (n = 603) were randomly divided into training and test groups with a ratio of 2:1 using the computer random number generator. Each monitoring indicator was assessed using t-test and chi-squared tests in the two groups, and the results confirmed that there was no statistically significant difference in each indicator between these two groups (*P*>0.05), as shown in Table 1. According to the revised Atlanta classification of acute pancreatitis 2012, patients with SAP accounted for 17.2%. Among 603 patients, 63.3% were male patients (382/603), with a mean age of 47.1 years. The overall mortality rate was 2.5% (15/603) in 30 days.

**Table 1:** Baseline characteristics of patients in the training and test samples.

Variable	training group	test group	*P* value
N	402	201	
Age(year)	46.41 ± 14.35	47.87 ± 14.55	0.246^a^
Male(%)	262 (65.5%)	118 (59.0%)	0.119^b^
Temperature	36.84 ± 0.65	36.91 ± 0.96	0.254^a^
Heart rate	94.68 ± 20.73	94.55 ± 21.90	0.942^a^
Respiratory rate (RR)	21.76 ± 4.01	21.84 ± 3.97	0.826^a^
Systolic blood pressure(mmHg)	129.46 ± 19.75	130.98 ± 21.17	0.388^a^
Blood glucose levels(mmol/l)	8.67 ± 4.60	9.58 ± 5.10	0.052^a^
White blood cell count(10*9/l)	11.92 ± 5.03	11.71 ± 4.75	0.622^a^
Hematocrit(%)	40.17 ± 6.04	39.60 ± 5.86	0.280^a^
Platelet(10*9/l(	171.62 ± 63.80	169.54 ± 76.56	0.731^a^
Total bilirubin(μmol/l)	29.21 ± 28.06	27.53 ± 22.11	0.462^a^
Aspartate transaminase(U/l)	73.22 ± 115.39	69.36 ± 102.87	0.691^a^
Alanine aminotransferase(U/l)	77.81 ± 140.60	79.20 ± 118.08	0.905^a^
Albumin(g/l)	38.05 ± 27.42	40.52 ± 31.08	0.513^a^
Lactate dehydrogenase(U/l)	314.00 ± 241.65	292.24 ± 245.50	0.420^a^
Blood urea nitrogen (mmol/l)	5.61 ± 4.33	6.17 ± 4.75	0.146^a^
Creatinine(μmol/l)	89.61 ± 112.74	81.32 ± 58.67	0.329^a^
Serum sodium (mmol/l)	138.07 ± 4.64	138.09 ± 4.16	0.970^a^
Potassium (mmol/l)	4.13 ± 0.67	4.10 ± 0.62	0.612^a^
Calcium(mmol/l)	2.05 ± 0.33	2.01 ± 0.33	0.162^a^
Triglyceride (mmol/l)	5.01 ± 7.09	5.49 ± 10.20	0.665^a^
C-reactive protein	36.6±63.2	36.1±73.5	0.552^a^
PH	7.35 ± 0.10	7.36 ± 0.07	0.462^a^
PaO_2_ (mmHg)	89.09 ± 29.51	89.53 ± 31.23	0.923^a^
PaCO_2_ (mmHg)	35.44 ± 9.24	36.95 ± 7.63	0.249^a^
Base excess (mmol/l)	-4.89 ± 6.76	-3.86 ± 4.30	0.297^a^
FIO_2_(%)	0.28 ± 0.02	0.28 ± 0.05	0.340^a^
Oxygenation index	315.96 ± 107.83	316.26 ± 103.05	0.987^a^
Prothrombin time(s)	14.45 ± 2.04	14.33 ± 1.84	0.590^a^
Fibrinogen(g/l)	5.30 ± 2.33	5.08 ± 2.22	0.438^a^
SAP(%)	68 (17.0%)	35 (17.5%)	0.878^b^

^a^t-test

^b^Chi-square test

All those data were measured in the first 12 hours after the patients were admission.

### Univariate and multivariate regression analyses

In the training group, regression analyses were performed to identify the presence of SAP, and the results found that there were significant differences in indicators including heart rate, RR, SBP, glucose, *et al*. between the SAP and non-SAP groups, as illustrated in Table 2. Next, the relevant e mentioned indicators were evaluated by multivariate regression analysis using the generalized linear models in the Empower-Stats, and the results are illustrated in Table 3. Finally, the four indicators were screened as predictive factors: each additional RR (OR = 1.15, [95% CI, 1.02–1.28]; *P* <0.01), every one unit increment of LDH (OR = 1.01, [95% CI, 1.01–1.01]; *P* <0.01), every one unit increment of creatinine (OR = 1.03, [95% CI, 1.01–1.04]; *P* <0.01), and every one unit increment of OI (OR = 0.98, [95% CI, 0.97–0.99]).

**Table 2:** Univariate analysis of predictive factors of severe acute pancreatitis (SAP) in the training sample.

Variable	Non-Sap	Sap	*P* value
N	334	68	
Age(year)	45.95 ± 14.09	48.68 ± 15.46	0.154^a^
Male(%)	212 (63.9%)	50 (73.5%)	0.126^b^
Etiology			0.297^b^
Gallstones	192(57.5%)	35(51.5%)	
Alcohol	62(18.6%)	15(22.1%)	
Triglyceride	50(15.0%)	13(19.1%)	
Post-ERCP	10(3.0%)	4(5.9%)	
Idiopathic	20(6.0%)	1(1.5%)	
Temperature	36.86 ± 0.64	36.74 ± 0.69	0.165^a^
Heart rate	91.37 ± 17.92	110.04 ± 25.61	<0.001^a^
Respiratory rate (RR)	21.09 ± 2.92	24.91 ± 6.31	<0.001^a^
Systolic blood pressure(mmHg)	128.37 ± 17.88	134.47 ± 26.38	0.021^a^
Blood glucose levels(mmol/l)	8.00 ± 3.28	11.45 ± 7.45	<0.001^a^
White blood cell count (10*9/l)	11.73 ± 4.86	12.83 ± 5.70	0.105^a^
Hematocrit(%)	39.77 ± 5.68	42.04 ± 7.26	0.005^a^
Platelet(10*9/l)	177.21 ± 64.17	145.19 ± 55.20	<0.001^a^
Total bilirubin(μmol/l)	28.43 ± 27.78	33.20 ± 29.33	0.210^a^
Aspartate transaminase(U/l)	70.81 ± 115.04	85.00 ± 117.24	0.360^a^
Alanine aminotransferase(U/l)	75.55 ± 111.82	89.00 ± 237.37	0.479^a^
Albumin(g/l)	40.80 ± 32.49	33.42 ± 14.70	0.140^a^
Lactate dehydrogenase(U/l)	249.58 ± 167.16	555.21 ± 316.72	<0.001^a^
Blood urea nitrogen (mmol/l)	4.66 ± 2.42	10.25 ± 7.53	<0.001^a^
Creatinine(μmol/l)	67.55 ± 24.61	197.33 ± 241.88	<0.001^a^
Serum sodium (mmol/l)	138.15 ± 4.31	137.69 ± 6.02	0.452^a^
Potassium (mmol/l)	4.04 ± 0.52	4.55 ± 1.06	<0.001^a^
Calcium(mmol/l)	2.11 ± 0.28	1.76 ± 0.39	<0.001^a^
Triglyceride (mmol/l)	4.24 ± 6.34	7.92 ± 8.97	0.014^a^
C-reactive protein	50.43±92.1	72.32±89.2	0.001^a^
PH	7.38 ± 0.07	7.32 ± 0.12	0.001^a^
PaO_2_(mmHg)	94.38 ± 30.80	82.96 ± 26.92	0.033^a^
PaCO_2_ (mmHg)	37.08 ± 7.66	33.52 ± 10.55	0.034^a^
Base excess (mmol/l)	-2.89 ± 4.79	-7.18 ± 7.92	<0.001^a^
FIO_2_(%)	0.28 ± 0.00	0.28 ± 0.04	<0.001^a^
Oxygenation index	340.99 ± 119.05	283.00 ± 81.15	0.009
Prothrombin time(s)	14.15 ± 1.60	15.32 ± 2.80	<0.001^a^
Fibrinogen(g/l)	5.02 ± 2.16	6.37 ± 2.66	0.004^a^

^a^t-test

^b^Chi-square test

**Table 3:** Multivariate regression analyses.

	*P*	OR	95%CI low	95%CI upp
Heart rate(bpm)	0.94	0.99	0.97	1.02
Respiratory rate (RR)	0.01	1.15	1.02	1.28
Lactate dehydrogenase	<0.01	1.01	1.01	1.01
Blood urea nitrogen (mmol/l)	0.94	0.99	0.85	1.15
Creatinine(μmol/l)	<0.01	1.03	1.01	1.04
Calcium(mmol/l)	0.36	0.42	0.06	2.75
Oxygenation index	<0.01	0.98	0.97	0.99

### Development of the decision tree model

**Fig 1:**
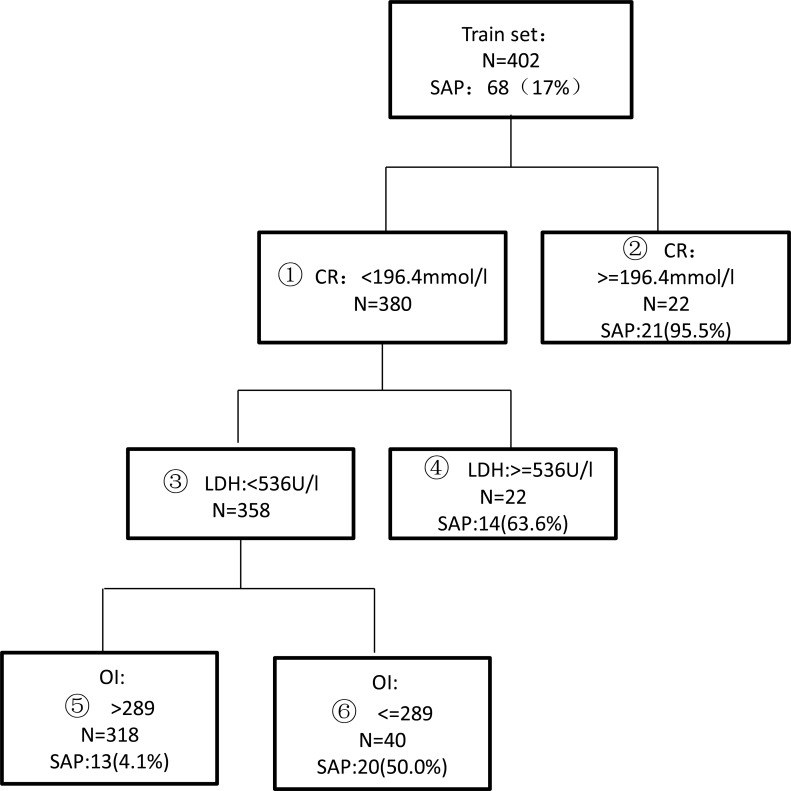
A decision tree model for the prediction of severe acute pancreatitis (SAP) generated by classification and regression tree (CART) analysis in the training set of 402 patients.

The above four indicators were analyzed using the decision tree method and SPSS 18.0 software. Several decision tree models were automatically generated by the system, and the best decision tree was selected according to the diagnostic sensitivity, specificity, accuracy and other indicators, as shown in Fig 1. Finally, creatinine, LDH, and OI were selected as the screening indicators, and the respective cut-off values were 196.4 μmol/L for creatinine, 536 U/L for LDH, and 289 for OI. The nodes were divided into two groups according to the possibility of the development of SAP. The high-risk group included node 2 (creatinine greater than or equal to 196.4 μmol/L), node 4 (creatinine smaller than 196.4 μmol/L, LDH greater than or equal to 536 U/L), and node 6 (creatinine smaller than 196.4 μmol/L, LDH smaller than 536 U/L, and OI smaller than 289). Overall, 64.5% (55/84) of the patients in the high-risk group developed SAP. The low-risk group included node 5 (creatinine smaller than 196.4 μmol/L and LDH smaller than 536 U/L, and OI greater than 289). Only 4.1% (13/318) of the patients from the low-risk group eventually developed SAP.

The evaluation index for the diagnosis of SAP using the decision tree model is illustrated in Table 4. The sensitivity, specificity, and overall accuracy in diagnosing SAP were 80.9%, 90.0%, and 88.5%, respectively.

**Table 4:** Diagnostic values of various predictors in the tree model.

Variable	Se(%)	Sp(%)	PPV(%)	NPV(%)	DA(%)
Cr(> = 196.4μmol/L)	30.9	99.7	95.5	87.6	88.0
LDH(> = 536U/L)	20.9	97.6	72.4	87.3	86.2
OI(< = 289)	52.9	93.1	61.0	90.7	86.3
Cr,LDH combined	51.5	97.3	79.5	90.7	89.5
Cr,LDH,OI combined	80.9	90.0	61.8	95.9	88.5

Cr: Creatinine, LDH: Lactate Dehydrogenase, BE: Base Excess, Se: Sensitivity, Sp Specificity, PPV: Positive predictive value, NPV: Negative Predictive Value, DA: Diagnostic Accuracy

### Validation of the decision tree model

**Fig 2:**
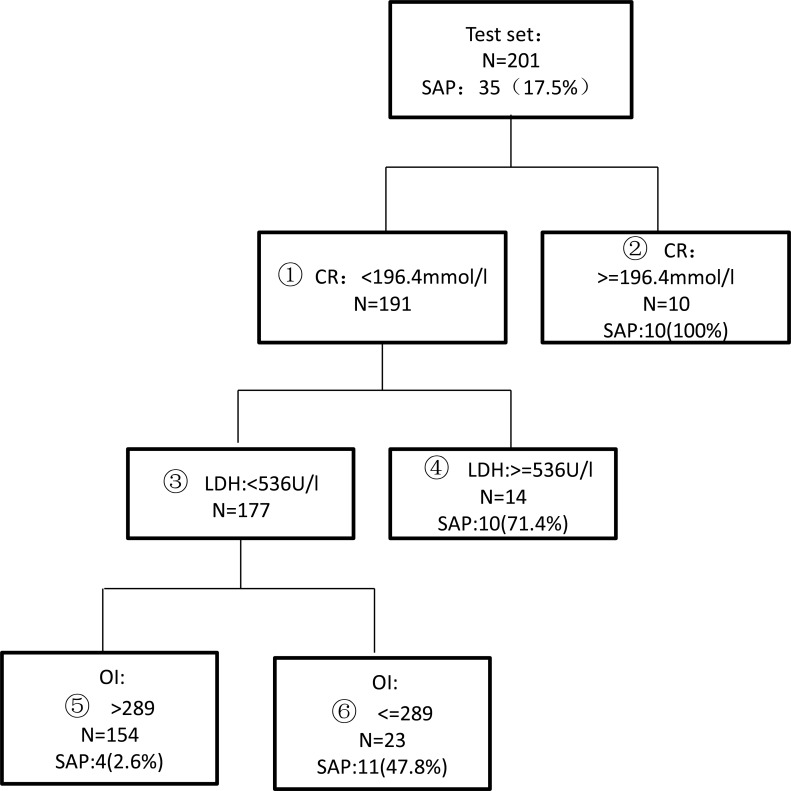
Validation of the test set of the tree model obtained by CART analysis.

**Fig 3:**
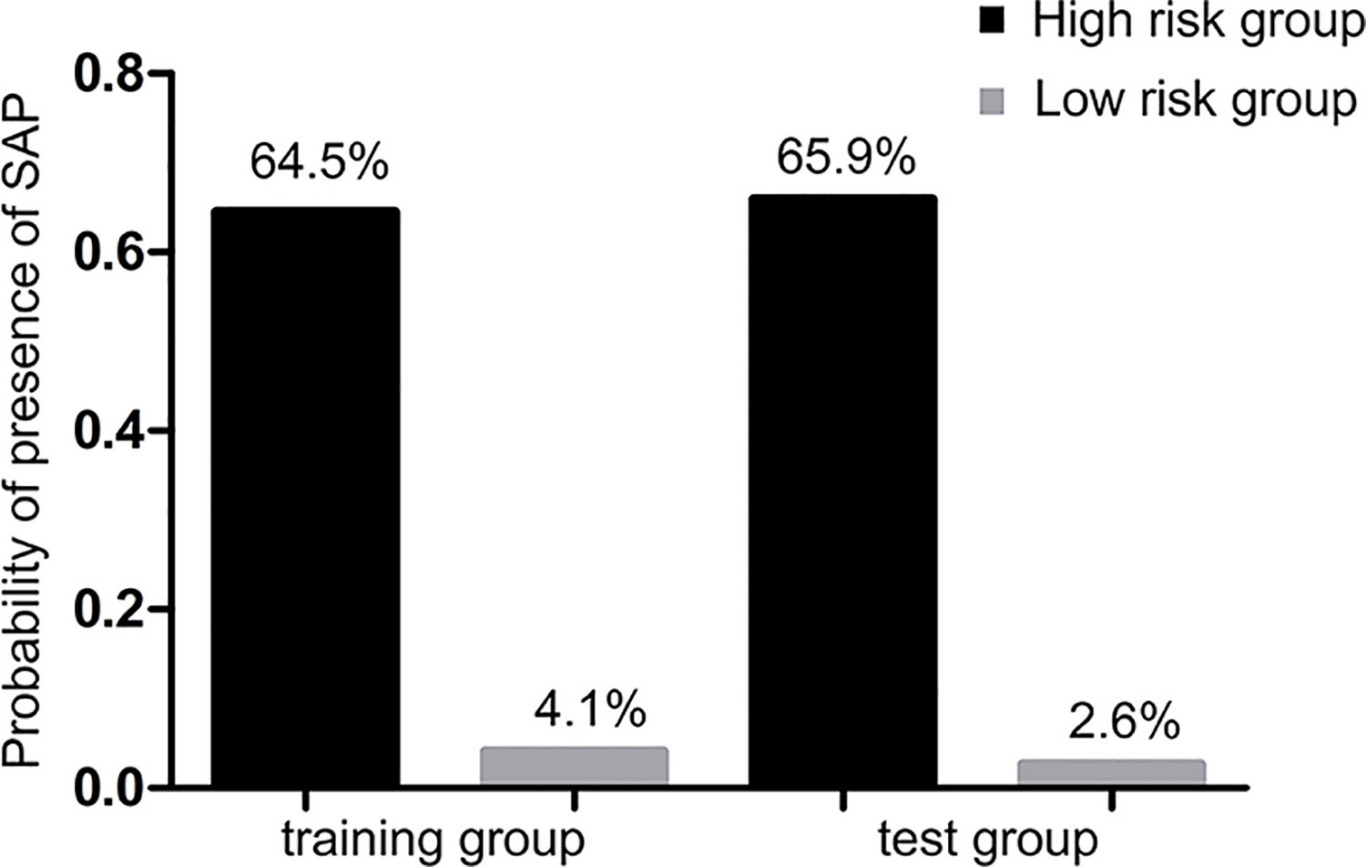
Patients stratified by the tree model in the training and the test groups.

The prediction of the decision tree model was verified using the data of the test group according to the range of the three indicators, as shown in Fig 2. The sensitivity and specificity of the decision model in the test group were 88.6% and 90.4%, respectively. The probabilities of SAP development in the high-risk and low-risk groups were 65.9% (31/47) and 2.6% (4/154), respectively, as shown in Fig 3.

**Fig 4:**
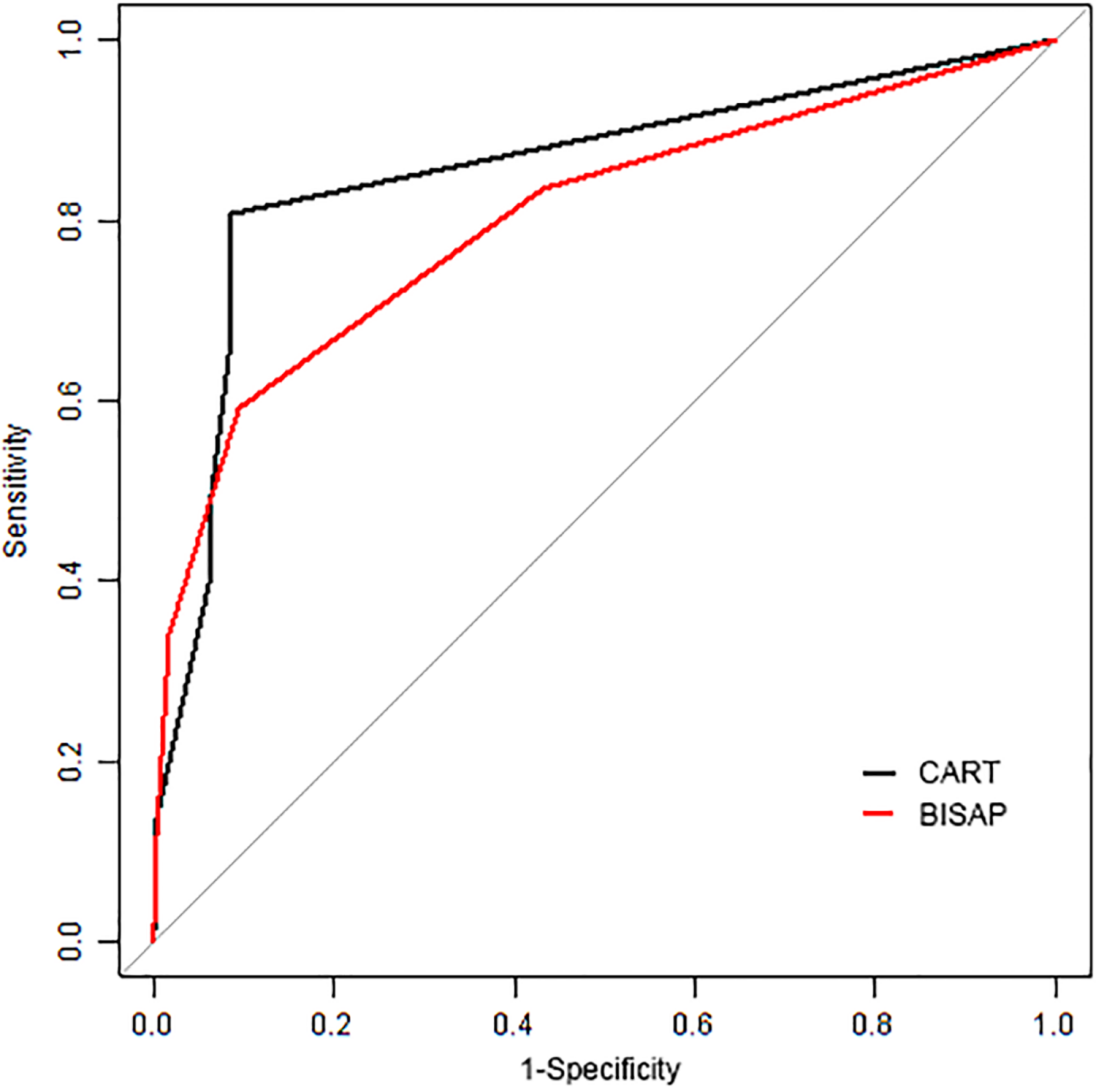
Receiver operating characteristic curves for classification and regression tree (CART model) and Bedside Index for Severity in Acute Pancreatitis(BISAP model).

The Bedside Index for Severity in Acute Pancreatitis (BISAP) [13]score was used to predict mortality and severe acute pancreatitis based on 1992 Atlanta criteria. Gao[18] reclassified the patients according to the revised Atlanta criteria and performed an meta-analysis. Therefore we performed receiver operating characteristic (ROC) analysis to compare those two different prediction models. As shown in Fig 4, the area under the curve (AUC) of BISAP was 0.802 (95%CI: 0.752–0.853), and the AUC of CART was 0.855 (95%CI: 0.814–0.896). There is no significant difference between those two predict models *P* = 0.098. However the value of cut-off point in CART is better than BISAP. When set BISAP score > = 2 as a predict criteria, the sensitivity was 59.2%, and the specificity was 90.6%. The sensitivity was 80.58%, and the specificity was 91.4% when employed all the three indicators as predict criteria in CART model.

## Discussion

In this retrospective study, of the patients with AP were admitted to our department, only 46.1% were admitted within 36 h after the onset of AP. Among 603 patients, 9 (1.5%) had heart failure, 131 (21.7%) had respiratory failure, and 38 (6.3%) had renal failure, and clarify that 103 patients were considered as SAP in the end. In the training group, univariate and multivariate regression analyses showed that there were significant differences in RR, creatinine, LDH, and OI and creatinine between the SAP and non-SAP groups. Ultimately, creatinine, LDH, and OI were selected as predictive indicators for the decision tree model.

Elevated creatinine levels are common in early stage of acute pancreatitis. A systemic inflammatory response and a high rate of catabolism in the body lead to significantly increased creatinine. Meanwhile, systemic capillary leak and reduced effective circulatory blood volume may activate the sympathetic nervous system, leading to contraction of renal arteries, and subsequent reduction of glomerular filtration rate, and increase of creatinine and BUN. In the previous several SAP predictive models, BUN was used as an independent prognostic factor [19,20], and it was believed that a decreased BUN level within 24 h of onset with sufficient fluid resuscitation promoted a better prognosis [13]. However, the present study data suggested that the BUN level was not a good predictor of SAP (*P* = 0.94)., whereasthe creatinine level offered a good predictive value (*P* <0.01). According to the distribution of serum creatinine, we found that once the creatinine was greater than 232 μmol/L, it would never return to the normal range within 48 h of onset in almost all the patients, and these patients could be identified as SAP in the early stage. To improve the diagnostic sensitivity of SAP, a creatinine level >196.4 μmol/L was selected as the determinant criteria. Twenty-two patients were diagnosed with SAP in the training group, among which 95.5% were ultimately identified as SAP. BUN could not be considered as a predictive indicator in this study, it might be related to the diagnostic criteria (Atlanta classification of acute pancreatitis 2012) used in the present study; however, in previous studies, Atlanta classification 1992 was used. In the previous reports, BUN greater than 25 mg/dl was considered as a predictive criterion [21]. However, in the present study, BUN was greater than 25 mg/dl (8.9 mmol/l) in 89 patients, among whom 49.3% (34/69) had a creatinine level smaller than 170 μmol/l that could not be diagnosed as renal failure. Interestingly, if considering a creatinine level greater than 170 μmol/l as the cut-off value, 38 patients met this criteria, of whom 89.5% (34/38) had BUN greater than 25 mg/dl. Thus, we think creatinine is more sensitive than BUN in identifying the severity of pancreatitis using renal failure, according the revised Atlanta criteria 2012.

LDH, a glycolytic enzyme, is present in the cytoplasm of all living cells, and can be found abundantly at higher concentrations in the heart, kidney, and skeletal muscles. In patients with SAP accompanied by organ dysfunction, blood LDH levels are significantly increased. ZIM et al. found that a severe LDH increase can be considered a predictive indicator of acute pancreatitis in the early mortality of patients [11]. According to the LDH different distribution between the two groups, we found that all patients with LDH > 727 U/L developed SAP. To improve the diagnostic sensitivity of SAP, LDH > 536 U/L was selected as the determinant factor, and 22 patients were diagnosed as SAP, of whom 63.6% (14/22) were eventually identified as SAP.

The OI represents the oxygenation index of lung tissues and represents the ratio of the partial pressure of arterial oxygen and concentration of inhaled oxygen. Early systemic inflammatory response syndrome (SIRS) may lead to acute lung injury. Meanwhile, pleural effusion and airway hyperreactivity reduce effective ventilation and lead to hypoxemia. Hence, the lung is the primary target organ for SIRS. Among all organ dysfunctions, pulmonary insufficiency accounts for the highest proportion. In the present study, considering the set conditions of creatinine < 196.4 mmol/l and LDH < 536 U/L, 40 patients were diagnosed as SAP with an OI ≤ 289, of whom 50% (20/40) were eventually identified as SAP.

Concerning the above mentioned conditions, the diagnostic sensitivity of SAP in the decision tree model had reached a relatively high satisfactory value of 88.6%, whereas the specificity and overall accuracy were 90.4% and 90.0%, respectively. According to the possibility of patients eventually developing SAP, the patients were divided into high-risk and low-risk groups. In the high-risk group, 64.5% of the patients eventually developed SAP, while only 4.1% of the patients in the low-risk group had developed to SAP. The accuracy was further confirmed in the test group; thus, the proposed model could well predict the severity of the disease at early stage.

In the present study, a decision tree model was developed to predict the SAP in patients with acute pancreatitis using three clinical indicators obtained within 12 h of admission, which could enable clinicians to choose more appropriate treatment for high-risk patients during a short period. We compared our predict model with BISAP predict model, and found there was no significant difference in AUC. But the best cut-off point in CART is more suitable than BISAP. Since SAP is a critical disease, it is important to detective SAP as more as possible. Therefore the sensitivity of the predict model is of vital importance. The sensitivity is higher in CART model when the specificity is similar between those two models.

It must be pointed out that the present study is a single-center retrospective study. There are some deficiencies of included indicators. We are verifying it in our clinic work. And the effectiveness of this decision tree model needs to be verified further in future prospective studies.

## Supporting Information

S1 File Relevant data underlying the findings described in manuscript. (XLSX)
